# First Draft Genome Sequences of Three Strains of *Francisella tularensis* subsp. *holarctica*, Isolated from Hares and a Tick in France

**DOI:** 10.1128/genomeA.00993-17

**Published:** 2017-11-09

**Authors:** Nora Madani, Philippe Giraud, Christiane Mendy, Cécile Colaneri, Emeline Cherchame, Moulay-Ali Cherfa, Céline Richomme, Anouk Decors, Guillaume Girault

**Affiliations:** a University Paris-Est, Anses, Bacterial Zoonoses Unit, Animal Health Laboratory, Maisons-Alfort, France; b Veterinary Laboratory of Pas-de-Calais, Arras, France; c Anses Nancy, Laboratory for Rabies and Wildlife, Malzéville, France; d National Hunting and Wildlife Agency, Research and Expertise Department, Le Perray en Yvelines, France

## Abstract

Here, we report the complete genome sequences of three strains of *Francisella tularensis* subsp.* holarctica* (11-789-5S, 11-935-13S, and 11-930-9S), isolated from brown hares and a tick during a tularemia outbreak in France, where tularemia is endemic.

## GENOME ANNOUNCEMENT

Tularemia is a zoonosis caused by *Francisella tularensis*. This bacterium is a Gram-negative, coccoid rod, nonmotile, and non-spore-forming organism that is an obligate aerobe with optimal growth at 37°C. Tularemia occurs naturally in lagomorphs and in rodents. A wide range of other mammals and several species of birds have also been reported to be infected (1). Two types of *F. tularensis* are recognized on the basis of their epidemiology and virulence in hosts. *Francisella tularensis* subsp.* tularensis* (type A) is associated with lagomorphs in North America. *Francisella tularensis* subsp.* holarctica* (type B) occurs mainly in aquatic rodents in North America and in hares and small rodents in Eurasia. Type B is water or arthropod borne and is less virulent to humans and rabbits than type A (2). Tularemia is a notifiable zoonosis in France and is listed as a potential bioterrorist weapon (3). Tularemia is endemic in France and occurs usually as sporadic cases. In winter 2011, we reported an outbreak of tularemia in brown hares near Habarcq in Pas-de-Calais, France. This outbreak was the first one in the area and was characterized by a high mortality rate in the local hare population (4).

Here, we present the complete genome sequences of three *F. tularensis* subsp. *holarctica* strains, two of which were isolated during the Habarcq outbreak from a hare (11-935-13S) and from a questing tick that was caught (11-789-5S). The third strain, 11-930-9S, was isolated in the same period from a sporadic case in a brown hare found dead in another area. These isolates were confirmed to be *F. tularensis* by both bacteriological and real-time PCR (5). All procedures were performed under biosafety level 3 conditions. Further, subtyping of *F. tularensis* colonies was performed by PCR targeting of the genomic region RD1 that allows differentiation between *F. tularensis* subsp. *tularensis* and *F. tularensis* subsp. *holarctica* (6).

Genomic DNA was extracted from each strain culture. Whole-genome sequencing was performed using the HiSeq 2000 system (Illumina, Inc.). Single-end reads of 100 bp in length were generated. The numbers of reads that passed the Illumina quality filters were 17,777,248 (11-930-9S), 17,270,587 (11-935-13S), and 17,687,454 (11-789-5S), for a total of 1.778 Gbp, 1.727 Gbp, and 1.769 Gbp, respectively. The data sets were *de novo* assembled using SPAdes 3.7.1 (7), with various k-mer sizes (21, 33, 55, and 77). The numbers of contigs generated that were >500 kb are 103, 107, and 106 for 11-789-5S, 11-930-9S, and 11-935-13S, respectively. The total lengths of the assemblies are 1,824,963 bp (11-789-5S, 235 contigs, 32.21% G+C content), 1,841,960 bp (11-930-9S, 275 contigs, 32.7% G+C content), and 1,852,368 bp (11-935-13S, 328 contigs, 32.24% G+C content). The largest contig obtained has a size of 87,580 bp (for the three strains), and the *N*_50_ value is 26,948 bp (for the three strains). The average coverages for the contigs were 204×, 205×, and 206× for 11-935-13S, 11-930-9S, and 11-789-5S, respectively.

The sequencing data could be compared with those of strains isolated from human cases of tularemia to study the epidemiological link between human and animal cases.

### Accession number(s).

The draft genome sequences for the three strains have been deposited at ENA/EMBL under the BioProject number PRJEB11276 (ERP012640) and the accession numbers CZDG02000001 to CZDG02000328 (11-935-13S), CZDH02000001 to CZDH02000275 (11-930-9S), and CZDI02000001 to CZDI02000235 (11-789-5S).
